# Tools for measuring client experiences and satisfaction with healthcare in low- and middle-income countries: a systematic review of measurement properties

**DOI:** 10.1186/s12913-023-09129-9

**Published:** 2023-02-09

**Authors:** Susan Banda, Nthanda Nkungula, Isabel Kazanga Chiumia, Jamie Rylance, Felix Limbani

**Affiliations:** 1 Malawi Liverpool Wellcome Trust Clinical Research Program, P.O. Box 30096, Chichiri, Blantyre 3, Malawi; 2 Health Economics and Policy Unity, Kamuzu University of Health Sciences, Blantyre, Malawi

**Keywords:** Systematic review, Client experiences, Client satisfaction; Patient-centered care, Healthcare quality, Tools, Instruments, Low- and middle-income countries

## Abstract

**Background:**

Perspectives of patients as clients on healthcare offer unique insights into the process and outcomes of care and can facilitate improvements in the quality of services. Differences in the tools used to measure these perspectives often reflect differences in the conceptualization of quality of care and personal experiences. This systematic review assesses the validity and reliability of instruments measuring client experiences and satisfaction with healthcare in low- and middle-income countries (LMICs).

**Methods:**

We performed a systematic search of studies published in PubMed, SCOPUS, and CINAHL. This review was reported according to the Preferred Reporting Items for Systematic Review and Meta-analysis (PRISMA) guidelines. Studies describing the development and psychometric properties of client experience and satisfaction with general health care were included in the review. Critical appraisal of study design was undertaken using the Appraisal tool for Cross-Sectional Studies (AXIS). The Consensus-based Standards for the Selection of Health Measurement Instruments (COSMIN) checklist and Terwee’s criteria were used to appraise the psychometric properties of the included studies. A narrative synthesis approach was used in the interpretation of the findings.

**Results:**

Of the 7470 records identified, 12 studies with 14 corresponding instruments met the inclusion criteria and were included in the final review. No study assessed all the psychometric properties highlighted by the COSMIN criteria. In most instruments, we found evidence that initial development work incorporated client participation. The most evaluated measurement properties were content validity, internal consistency, and structural validity. Measurement error and responsiveness were not reported in any study.

**Conclusion:**

Reliability and validity should be considered important elements when choosing or developing an instrument for professionals seeking an effective instrument for use within the population. Our review identified limitations in the psychometric properties of patient experience and satisfaction instruments, and none met all methodological quality standards. Future studies should focus on further developing and testing available measures for their effectiveness in clinical practice. Furthermore, the development of new instruments should incorporate clients' views and be rigorously tested or validated in studies with high methodological quality.

**Trial registration:**

CRD42020150438.

**Supplementary Information:**

The online version contains supplementary material available at 10.1186/s12913-023-09129-9.

## Introduction

Improvements in the quality of healthcare are crucial in ensuring progress towards the Sustainable Development Goals (SDGs) and Universal Health Coverage (UHC) by 2030 [1]. Healthcare should be “compassionate, empathetic, and responsive to the needs, values, and preferences of all individuals and ensure patient values guide all clinical decisions” [2]. Measurement of patients’ experiences of care is therefore crucial for achieving high-quality health services [2, 3]. Good patient experiences are associated with improved health outcomes, including better health care utilization, higher adherence to treatment, and lower resource use in secondary care [4, 5].

Patient-reported quality is an important component of healthcare quality and health service evaluation—it reflects the dimensions of quality relevant to the client [4, 5]. Patients offer a complementary perspective to that of healthcare providers, providing unique information and insights into both the humanity of care (such as dignity and respect, privacy, effective communication, emotional support, waiting time, delays, and cleanliness of facilities) and the effectiveness of health care [6–8]. Although predetermined definitions of quality are also used [9], successful elucidation of experience should ideally directly measure what matters most to patients [10, 11].

Studies have shown that patient experiences are related to patient satisfaction [12]. A key challenge with satisfaction surveys is that they often report high satisfaction, even in low-income settings with limited resources and relatively low-quality services [7, 13, 14], which limits the utility of satisfaction data as a quality measure [7, 10] and challenges the usefulness of satisfaction surveys in quality improvement work, thus calling for a more robust and multi-faceted approach [15]. Complementary approaches examine patients’ experiences within different domains of healthcare [6, 7].

Various methods may be used to assess client experiences and satisfaction with healthcare. These include qualitative and quantitative methods, as well as interviews, focus group discussions (FGDs), patient forums, formal complaints, observations, and informal feedback through patient advocacy groups [11]. Quantitative methods may be expedient but may fail to capture the multidimensional quality. For example, patients who said they would recommend a hospital nevertheless indicated problems in all dimensions of an experience questionnaire [16]. Thus, it is imperative to complement quantitative with qualitative methods to accurately capture how patients define and perceive their care [9, 17].

Manary et al. highlighted three concerns with patient-reported measures [18]. Firstly, satisfaction measures are subjective indications of how well patient expectations are met and therefore influenced by factors unrelated to health care [8, 18]. Similarly, current health status affects responses and may not be directly related to the quality of care. Thirdly, patient responses may be skewed by the most immediate experience, i.e., the receipt of specific medications [18]. Subjectivity can be reduced by focusing on validated measures, careful phrasing of questions and response choices, and using questions that assess aspects of care that were or were not provided during interactions with providers and the health care system [10, 19].

Standardized tools for monitoring and reporting patients’ perspectives derive mostly from high-income countries [3]. Instruments should be chosen according to both their psychometric properties and the purpose and context of the survey [20]. This highlights the need for countries to develop context-specific, valid, and reliable instruments to accurately capture aspects of care important to the population they serve.

Despite some existing systematic reviews that assessed the psychometric properties of patient experience measures of care across a range of settings and diseases [20–22], we found none for general health care in low- and middle-income countries (LMICs). This review aims to assess the validity and reliability of instruments measuring client experience and satisfaction with healthcare developed for use in LMICs.

## Methods

A protocol for this review was registered with the International Prospective Register of Ongoing Systematic Reviews (PROSPERO) (registration number: CRD42020150438). This systematic review was reported following the Preferred Reporting Items for Systematic Reviews and Meta-Analysis (PRISMA) guidelines [23].

### Study searches and eligibility criteria

We conducted a comprehensive literature search of the following electronic databases: PubMed (from 1946–November 7, 2019), CINAHL (EBSCOHost) (from 1982–November 7, 2019), and SCOPUS (Elsevier) (from 1966–November 7, 2019). The search was conducted using a combination of keywords, Boolean operators, and MeSH terms. Free text terms and MeSH terms derived from or related to the selected keywords were also included in the search strategies. The following five main groups of search terms were used to develop the search strategy: (i) tool, instrument, survey, questionnaire, or scale (ii) patient or client (iii) patient satisfaction, patient experience, client satisfaction, client experience, or patient-centered care (iv) health care quality or health care assessment (v) LMICs—We modified the LMICs search filter from a previous review [24] and included countries classified as LMICs by the World Bank in 2019 [25]. Search results were limited to the English language for all the databases. The search strategy was initially created for PubMed and subsequently modified to meet specific search requirements for the additional databases. The Scopus search strategy excluded some high-income countries (see Additional file 1 for the complete search strategy used in each database). An update of the search was conducted for additional publications on November 12, 2021. The updated search was performed across all databases using the same search terms as the initial search. The reference lists of eligible articles were reviewed for additional literature. All records were exported to Mendeley (version 1.19.8) (https://www.mendeley.com/search/) for reference management and the removal of duplicates.

Studies were eligible for inclusion if they were peer-reviewed English-language articles that examined theoretical or conceptual development, psychometric properties, or the utility of instruments measuring patient experience or satisfaction with hospital-based health care (either inpatient or outpatient care). Studies were excluded if they derived from pediatric populations or high-income countries; were purely qualitative; were specific to a medical condition or procedure; or reported only Patient-reported outcome measures (PROMs).

### Study selection

Titles and abstracts were independently screened by two reviewers (SB and NN), utilizing the eligibility criteria. Full-text copies of selected articles were retrieved for detailed examination. Articles meeting the inclusion criteria underwent data extraction and quality assessment. Disagreements between the reviewers were discussed to achieve consensus.

### Data extraction, quality assessment, and analysis

Two reviewers (SB and NN) independently extracted data from the most recent version of the instrument using a pre-designed data extraction template, which included an assessment of: study characteristics; author; publication year; setting; country of origin; mode of administration; number of items; dimensions of patient satisfaction or experience; sample size; and psychometric properties.

The quality assessment involved two reviewers (SB and NN). The Appraisal tool for Cross-Sectional Studies (AXIS) was used to appraise bias in study design. AXIS comprises 20 questions assessing the quality of study design, including aims and objectives, sample size justification, methods, and presentation of results [26]. Quantitative thresholds for high-quality studies have not been established; we consider total scores of > 15 as high quality, 10–15 as moderate, and < 10 as poor quality [27].

The validity and reliability of the included instruments were assessed using the Consensus-based Standards for the Selection of Health Measurement Instruments (COSMIN) [28]. This tool assesses the quality of each measurement property investigated in a study according to internal consistency, reliability (test–retest), measurement error, content validity (face validity), structural validity, hypothesis testing, criterion validity, cross-cultural validity, and responsiveness. Additional file 2 provides definitions of the measurement properties. Each property is classified as "excellent", "good", "fair", or "poor". For each property, the score is determined by the lowest rating on the related checklist criteria [29]. In addition, Terwee’s criteria was used to evaluate the quality of each measurement property. Based on this criteria, each property was scored using a four-point rating scale: positive ( +), indeterminate (?), negative ( −), and no information available (0). The criteria is presented in Additional file 2 [30].

Discrepancies between reviewers at the data extraction and quality assessment stages were resolved by consensus through detailed discussion. We analyzed included studies using narrative synthesis and a tabular summary of key characteristics.

## Results

**Fig. 1:**
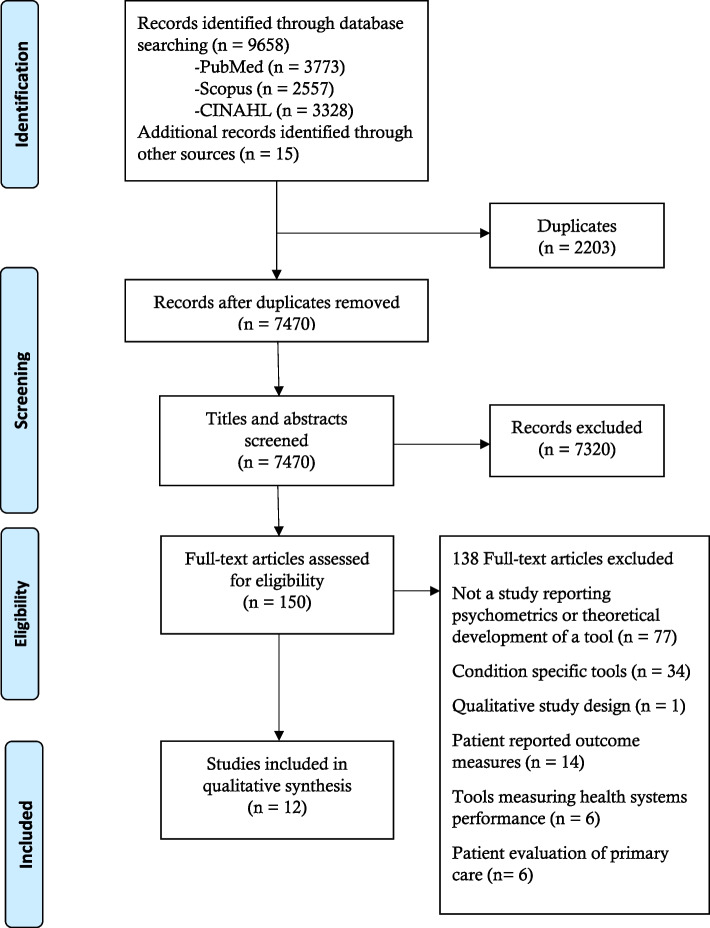
PRISMA flow diagram representing search and selection of studies

A total of 9658 records were obtained from the electronic search, and an additional 15 records were obtained from reference checking of relevant articles. After the removal of duplicates, a total of 7470 articles were screened. The full texts of 150 articles were assessed against the defined exclusion and inclusion criteria, of which 12 were analyzed (see Fig. 1).

### Characteristics of the included studies

Within 12 studies, 14 instruments were examined (Table 1). The studies were conducted in China [31–34], India [35, 36], Ethiopia [37], Hong Kong [38], Iran [39], Lebanon [40], Egypt [41], and Mexico [42]. Seven instruments measured patient experience with health care [32, 34, 35, 37, 38], while others measured patient satisfaction [31, 33, 39–42]. Sample sizes ranged from 230 to 6640 participants (see Table 1).

**Table 1:** Characteristics of studies

Author Year	Instrument	Setting	Number of Items	Sample Size	Mode of Administration	Dimensions	Timing of Administration
Wei et al. (2015) [31]	Chinese-Outpatient Satisfaction Questionnaire (CH-OPSQ)	46 hospitals China	19	5151	Self-administered	1. Waiting time 2. Service attitude 3. Quality of medical care 4. Quality of special services 5. Quality of environment 6. Global assessment	After medical procedures
Hu et al. (2017) [32]	Outpatient Experience Questionnaire (OPEQ)	6 public hospitals China	28	600	Interview administered	1.Physical environment 2. Doctor-patient communication 3. Healthy information 4. Medical expenses 5. Short time outcome 6. General satisfaction	After completion of an outpatient visit
Wong et al. (2015) [38]	Short-form of the Hong Kong Inpatient Experience Questionnaire (SF-HKIEQ)	25 public hospitals Hong kong	18	516	Telephone administered	1. Hospital staff 2. Patient care and treatment 3. Information on leaving hospital 4. Overall impression	48 h to 1 month after discharge
Wei et al. (2015) [33]	Inpatient Satisfaction Questionnaire (IPSQ)	3 hospitals China	28	6640	Self-administered	1. Waiting time 2. Service attitude 3. Medical care quality 4. Special service quality 5. Environment quality 6. Global assessment	During hospitalization, 3 days after admission
Wang et al. (2021) [34]	Outpatient Patient-Reported Experience Measure for Care in Chinese Hospitals (OPREM-CCH)	25 public hospitals China	22	2293	Interview administered	1. Communication and information 2. Professional competence 3. Medical costs 4.Efficiency 5.Hospital recommendations	After completion of an outpatient visit
Wang et al. (2021) [34]	Inpatient Patient-Reported Experience Measure for Care in Chinese Hospitals (IPREM-CCH)	25 public hospitals China	19	1510	Interview administered	1. Communication and information 2. Professional competence 3. Medical costs 4.Efficiency 5.Health outcomes 6.Hospital recommendations	During hospitalization, immediately before discharge
Webster et al. (2011) [37]	Patient Experiences with Inpatient Health care (I-PAHC)	8 health facilities Ethiopia	25	230	Interview administered	1.Communication with nurses 2.Communication with doctors 3. Physical environment 4. Pain management 5. Medication and symptom communication	During hospitalization, > 1 day after admission
Webster et al. (2011) [37]	Patient Assessment of Health care for outpatient Care (O-PAHC)	8 health facilities Ethiopia	23	486	Interview administered	1. Communication with nurses 2. Communication with doctors 3. Physical environment 4. Medication communication	After receiving care
Rao et al. (2006) [35]	Patient Perceptions on Quality (PPQ)	54 health facilities India	16	1837	Interview administered	1. Medicine availability 2. Medical information 3. Staff behavior 4. Doctor behavior 5. Hospital infrastructure	1) After receiving care (Outpatients) 2) During hospitalization, > 1 day after admission (Inpatients)
Goel et al. (2014) [36]	The North India Outpatient Department Satisfaction Scale (NIOPDSS)	6 hospitals India	17	1200	Mixed Interview administered Mail survey	1. Location 2. Administration 3. Waiting area 4. Pharmacy 5. Physicians 6. Basic facilities	After exiting the health facility
Arab et al. (2014) [39]	Persia Inpatient Satisfaction Questionnaire (PISQ)	6 hospitals Iran	52	400	Interview administered	1. Doctor-Patient communication 2. Nursing care 3. Convenience 4. Visitors 5. Cleanliness 6. Cost 7. General satisfaction	During hospitalization, at discharge
Zaghloul AA (2001) [41]	Patient satisfaction scale (PSC)	4 primary health centers Egypt	20	227	Interview administered	1. Art of care 2. Technical management 3. Accessibility 4. Structure	After completion of an outpatient visit
Kouatly et al. (2015) [40]	Patient Satisfaction Survey (PSS) -Middle East	1 hospital Lebanon	22	1339	Telephone administered	1. Care from nurses 2. Care from physicians 3. Admission and discharge 4. Hospital environment 5. Food services	After discharge
Garcia-Galicia et al. (2020) [42]	Outpatient department user satisfaction rapid scale (ERSaPaCE)	1 hospital Mexico	10	200	Self -administered	1. Cleanliness 2.Treatment by nursing staff 3. Treatment by medical staff 4.Information by the doctor 5.Assistance at the medical records department 6.Assistance at the pharmacy 7.Waiting time 8.General impression	Not specified

Most instruments were administered through face-to-face interviews (*n* = 9), others by self-completion (*n* = 3), telephone (*n* = 2), and mail survey (*n* = 1). The number of included items varied from 16 to 52, exclusive of demographic information. The timing of administration varied between instruments designed for inpatient and outpatient care. Exit interviews were conducted for all instruments tailored for outpatient care. The timing differed for inpatient care, from surveys being conducted during admission [33, 37], some on discharge [35, 39, 40], to several months after hospitalization [38]. Only five studies reported time for completing surveys, which ranged from within 5 to 15 min.

The number of dimensions in the instruments ranged from 4 to 8. Most instruments covered similar dimensions of timeliness, accessibility, environment, and facilities or basic amenities. All instruments included dimensions of communication and interpersonal components of care. Some encompassed aspects of pain management [37, 40], medication availability [35, 36], and technical competence of providers [34, 41]. Other instruments assessed overall satisfaction or the general impression of health services [31–33, 38, 39, 42]. One, a patient satisfaction survey, asked about the quality of food offered to inpatients [40]. Some instruments were structured around patients’ perceptions of quality of care [31, 41], while others focused on patient journeys from hospital admission to discharge (or entry to exit) [33, 35]. Contextual differences were reflected in the content of some instruments, e.g., the inclusion of aspects of payment-for-care or affordability [32, 34, 39].

### Quality assessment

All studies met the majority of AXIS tool criteria (see Additional file 3). The quality of studies was high in 9 (score > 15) and moderate in 3 (score 10–15). Across all studies, 10 of the AXIS criteria were consistently met. Major limitations in studies not meeting AXIS criteria were related to a lack of sample size justification and measures to address, categorize, or describe non-responders.

### Psychometric properties

An overview of the results of the COSMIN appraisal and quality rating of measurement properties is presented in Additional files 4 and 5, respectively. All instruments assessed content validity through a combination of literature review; consultation with experts, medical personnel, or the target population; cognitive interviews, and pilot testing of items. Scores for content validity were classified as excellent except for three instruments with poor ratings where it could not be ascertained if the target population was involved in the development process [36, 41, 42]. Structural validity, which assesses the degree to which scores of an instrument adequately reflect the dimensions of the construct being measured [30], was investigated in twelve instruments through confirmatory or principal component factor analysis [31–33, 35, 36, 41]. Seven instruments had a positive rating as factor analysis explained 50% of the variance [31–33, 35, 36, 41], whereas five had an indeterminate score as investigators did not report the variance explained by factors [34, 37, 39].

Hypothesis testing was presented in seven instruments. Four instruments had an excellent rating as hypotheses were set a priori [34, 37], whilst two had a fair rating [32, 33]. One instrument was classified as poor quality because no information was reported on the measurement properties of the comparator instrument [42]. Criterion validity was reported in three instruments. One instrument compared a short with its original longer version and had a positive rating as the correlation with the chosen gold standard was greater than 0.70 [38]. Two instruments compared scores with an outcome variable assessed at the same time and had negative ratings as correlations were less than 0.70 [34]. For cross-cultural validity, four instruments were translated but without documented testing of the translation; hence, they had an indeterminate rating [35, 37, 40]. None of the studies reported an evaluation of responsiveness, which is the ability of an instrument to detect changes over time [30].

Internal consistency was the most commonly reported indicator of reliability. Cronbach’s alpha, the most common measure of internal consistency, was reported in most studies. Terwee’s criteria consider a Cronbach’s estimate of between ≥ 0.70 and < 0.95 as adequate or good. A low estimate (< 0.70) indicates a lack of correlation between items in an instrument, making summarizing items unjustified. A high estimate (≥ 0.95) indicates high correlations among items in an instrument, e.g., redundancy of items [30]. Nine instruments had a positive internal consistency rating as subscales met the requisite Cronbach alpha threshold (see Additional file 2) [31–35, 38, 41], while five were classified as negative [37, 39, 40, 42]. Three studies reported evidence of test–retest reliability. One study had a fair COSMIN rating as no information was provided to ascertain if test conditions for both measurements were similar [38]. Two studies had poor ratings as test conditions were different [36, 42]. Measurement error was not reported in any studies.

## Discussion

This systematic review identified and appraised the psychometric properties of tools measuring client experience and satisfaction with health care developed for use in LMICs. Evaluating 14 instruments within 12 studies, none had a complete assessment of all relevant measurement properties. There was a general lack of evidence for the appraisal of most measurement properties, due either to incomplete reporting or poor-quality methodology. None of the studies reported measurement error and responsiveness.

Content validity assesses whether the content of an instrument reflects the construct to be measured [30]. It is regarded as the first measurement property to consider when selecting an instrument [43]. Our results show that only content validity and conceptual development were assessed fully in most of the included instruments. Item generation is a crucial step in the development of an instrument. When done correctly, it ensures that items of an instrument accurately and comprehensively cover the construct measured [44]. In most studies, clients or patients participated in item generation to determine what quality of care means to clients of health care services, which is necessary for the elusive and evolving concept of patient-centered care [3]. It is crucial to note that what matters to clients varies in different settings, hence, studies of cross-cultural validity are necessary if these instruments are used in other countries [21, 45].

Other frequently reported measurement properties were internal consistency and structural validity. The methodological quality of other properties, i.e., reliability, cross-cultural validity, and criterion validity, were generally fair to poor. This is likely due to the conservative nature of the COSMIN checklist, as scoring requires that overall ratings for each measurement property assessed be given according to the lowest score assigned over multiple criteria [29]. It is important to note that some COSMIN elements, such as cross-cultural validity, may not apply to all studies.

Criterion validity, which is considered when an instrument is compared with a gold standard, was reported in only three instruments. This may be because some authors lacked comparator instruments, as there are no gold standard instruments for either patient experience or satisfaction measures. According to COSMIN guidelines, a gold standard instrument for health-related patient-reported outcomes is generally impossible to find. For criterion validity, a long version of a shortened instrument can be considered a gold standard. However, if an instrument is considered a gold standard, studies comparing tools to this particular instrument provide evidence for criterion validity [46, 47].

From a policy and practice viewpoint, for a measure to be used to benchmark performance or evaluate interventions, it is vital to understand its ability to detect change in the concept being measured [48, 49]. Our results indicated that none of the studies assessed responsiveness. This is crucial as patient experience and satisfaction measures are used to guide quality improvement work in the health care system [4, 5]. Our results are consistent with similar systematic reviews which reported a lack of testing for instrument responsiveness [20, 21].

Our findings demonstrated that most instruments utilized dimensions that were linked to elements of patient-centered care. Across instruments, common domains were communication or interpersonal aspects of care, followed by physical comfort. Although the instruments reported a wide range of dimensions, certain aspects of care, such as coordination and continuity, remain under-reported. These are important dimensions that can influence the quality of care offered to clients, as poor coordination or continuity leads to clients receiving fragmented care, often with suboptimal outcomes and a risk of harm due to inadequate communication or sharing of information from providers and duplication of interventions [50]. As the desire to practice patient-centered care has gained prominence, it is crucial to consider the Institute of Medicine’s (IOM) dimensions of patient-centered care, which include: respect for patient's values, preferences, and expressed needs; coordination and integration of care; information, communication, and education; physical comfort; emotional support; and involvement of family and friends when developing patient experience and satisfaction measures [2].

Importantly, although patient experience and satisfaction are related, they are distinct concepts. Patient experience measures elicit feedback from patients regarding what happened before, during, and after interactions with the health care system [51, 52], whereas patient satisfaction involves evaluation of care provided relative to needs and expectations. Hence, patient satisfaction is an outcome of their experience [10]. Despite their differences, both measures are used to benchmark hospitals' performance, monitor health care quality, and assess the effectiveness of interventions [53].

The collection of client experience or satisfaction data has become an important part of the drive towards holistic patient-centered care [4]. It is a fundamental step towards improving the quality of care and health service evaluation [54, 55]. It is crucial to note that implementing multiple interventions, sustained over time, is required to attain significant improvements in health care [19]. Therefore, incorporating clients’ perspectives into quality improvement, coupled with other performance indicators and health outcomes, allows health systems to deliver quality health care and be accountable to the people they serve [56, 57].

### Study limitations

Our systematic review was limited by excluding grey literature and non-English articles. Missing values or insufficient reporting of psychometric properties, i.e., test–retest reliability, internal consistency, or cross-cultural validity, influenced the ratings regarding the adequacy of psychometric measures, and we did not contact authors for unpublished information. Our review is not a traditional description of diagnostic test accuracy; hence, the PRISMA guidelines were chosen over the Preferred Reporting Items for Systematic Reviews and Meta-Analysis of Diagnostic Test Accuracy Studies (PRISMA-DTA) guidelines. Nonetheless, we have been as comprehensive as possible to ensure important details are included using the PRISMA guidelines. Further, our systematic review used a broad search strategy to identify relevant evidence on tools measuring client experience and satisfaction in LMICs. This review is limited by the paucity of available data, which is particularly acute for sub-Saharan Africa.

### Implications for policy, practice, and future research

The use of valid and reliable instruments is key to providing accurate information to inform service delivery. The review identified limitations in the validity and reliability of patient experience and satisfaction measures developed for use in LMICs. Psychometric inadequacies and/or incomplete reporting of measurement properties indicate that further development and testing of these measures or validation of previous measures developed elsewhere for use in this region is required. Future research should emphasize the evaluation of psychometric properties based on the intended use of the measure (i.e., responsiveness for instruments used to track changes over time and measurement error to establish the clinical relevance of patient experience and satisfaction data used in health service evaluation) [22]. Importantly, instruments translated or used in different cultures, languages, populations, or settings should be assessed for cross-cultural validity to ensure that they measure the same concept as the original [58].

Instrument development should be based on good theoretical development, with item generation incorporating clients’ views. Qualitative feedback, including cognitive interviews and focus group discussions with the target population, is required to improve an instrument's content validity. We recommend applying standards such as the COSMIN checklist [28, 29] and Terwee’s criteria [30] when conducting studies of the psychometric properties of these instruments to fully understand their strengths and weaknesses.

Although patient experience and satisfaction have been widely studied in healthcare, gold-standard instruments have not been established [20, 59, 60]. Researchers and professionals need to be aware that the choice of an instrument depends on several factors, including the exact purpose of assessment, the target population, the setting, and available resources. Furthermore, the amount of time required to complete surveys is a crucial element to consider when selecting an instrument for use in either research, routine use, or quality improvement. The increased precision provided by more items may be balanced against the time saved by shorter instruments, which often facilitate a good response rate [60, 61].

## Conclusion

Patient experience and satisfaction measures are widely recognized as indicators of healthcare quality as they provide information on potential areas for improvement in healthcare delivery [2, 55]. For professionals seeking an effective instrument that produces credible results for use within the population, reliability and validity should be considered important elements when choosing or developing an instrument. Various tools measuring patient experience and satisfaction with general health care in LMICs are available, but our review identified limitations in their psychometric properties and none met all methodological quality standards. Therefore, based on our findings, we recommend that future studies focus on further development and testing of available measures. In addition, the development of new instruments should incorporate qualitative input from clients and be rigorously tested or validated in studies with high methodological quality. This systematic review may inform health care managers, researchers, clinicians, and policymakers when selecting or developing appropriate tools to assess experience or satisfaction with health care.

## Supplementary Information

**Additional file 1.** Database search strategies. The databases were accessed through Research4Life (https://portal.research4life.org/content/databases).

**Additional file 2.** Measurement property definitions and appraisal parameters (Terwee et al. 2007).

**Additional file 3.** Axis appraisal results.

**Additional file 4.** COSMIN quality assessment results.

**Additional file 5.** Terwee’s criteria quality assessment results.

## Data Availability

All data generated or analysed during this study is included in this article (and its additional files).

## Abbreviations

AXIS: Appraisal tool for Cross-Sectional Studies
CH-OPSQ: Chinese Outpatient Satisfaction Questionnaire
COSMIN: Consensus-based Standards for the Selection of Health Measurement Instruments
ERSaPaCE: Outpatient department user satisfaction rapid scale
FDGs: Focus group discussions
PROSPERO: International Prospective Register of Ongoing Systematic Reviews
IOM: Institute of Medicine
I-PAHC: Patient experiences with inpatient health care
IPREM-CCH: Inpatient Patient-Reported Experience Measure for Care in Chinese Hospitals
IPSQ: Inpatient Satisfaction Questionnaire
LMICs: Low-and middle-income countries
NIOPDSS: The North India Outpatient Department Satisfaction Scale
O-PAHC: Patient assessment of health care for outpatient care
OPEQ: Outpatient Experience Questionnaire
OPREM-CCH: Outpatient Patient-Reported Experience Measure for Care in Chinese Hospitals
PISQ: Persia Inpatient Satisfaction questionnaire
PPQ: Patient Perceptions on Quality
PRISMA: Preferred Reporting Items for Systematic Reviews and Meta-Analysis
PROMs: Patient-reported outcome measures
PSC: Patient satisfaction scale
PSS: Patient Satisfaction Survey
SDGs: Sustainable Development Goals
SF-HKIEQ: Short-form of the Hong Kong Inpatient Experience Questionnaire
UHC: Universal Health Coverage
