# Supplementary material for: Tools for measuring client experiences and satisfaction with healthcare in low- and middle-income countries: a systematic review of measurement properties
Source: BMC Health Serv Res. 2023 Feb 9;23:133. doi: 10.1186/s12913-023-09129-9 (PMC9909903; doi:10.1186/s12913-023-09129-9)
Supplement: Supplementary file 1 — Additional file 1. Database search strategies. The databases were accessed through Research4Life (https://portal.research4life.org/content/databases). [file 12913_2023_9129_MOESM1_ESM.docx]

**Additional file 1: Database search strategies**

The databases were accessed through Research4Life (<https://portal.research4life.org/content/databases>)

| 1. PubMed |
| --- |
| 1. ((((((((((((tool*[Title/Abstract]) OR surve*[Title/Abstract]) OR question*[Title/Abstract]) OR measur*[Title/Abstract]) OR instrument*[Title/Abstract]) OR scal*[Title/Abstract])  2. (Surveys and Questionnaires[MeSH Terms])) OR Health Care Surveys[MeSH Terms]))  3. (((Patients[MeSH Terms]) OR client*[Title/Abstract]) OR patient*[Title/Abstract]))  4. ((((((((((((("Patient Preference"[MeSH Terms]) OR "Patient Outcome Assessment"[MeSH Terms]) OR "Patient-Centered Care"[MeSH Terms]) OR "Patient Satisfaction"[MeSH Terms]) OR patient preference[Title/Abstract]) OR "Patient-Centered Care"[Title/Abstract]) OR "patient experience*"[Title/Abstract]) OR " patient satisfaction"[Title/Abstract]) OR "patient perception*"[Title/Abstract]) OR "patient opinion*"[Title/Abstract]) OR " patient view*"[Title/Abstract]) OR "responsiveness"[Title/Abstract]) OR "service delivery"[Title/Abstract]))  5. ((((((("Delivery of Health Care"[Mesh] OR "Quality Indicators, Health Care"[Mesh] OR "Health Care Evaluation Mechanisms"[Mesh] OR "Process Assessment (Health Care)"[Mesh] OR "Outcome Assessment (Health Care)"[Mesh] OR "Quality of Health Care"[Mesh])) OR "health care quality"[Title/Abstract]) OR "Delivery of Health Care"[Title/Abstract]) OR "Outcome Assessment (Health Care)"[Title/Abstract]) OR " Health care evaluation mechanism*"[Title/Abstract]) OR "Quality Indicators,Health Care"[Title/Abstract]))  6.(((((((((((((((((((((((((((((((((((((((((((((((((((((((((((((((((((((((((((((((((((((((((((((((((((((((((((((((((((((((((((((((((((((((((((((((((((((((((((((((((("Developing Countries"[Mesh]) OR "low income countr*"[Title/Abstract]) OR "middle income countr*"[Title/Abstract]) OR " low middle income countr*"[Title/Abstract]) OR "Poor countr*"[Title/Abstract]) OR " Northern Africa"[Title/Abstract]) OR "South of the Sahara Africa"[Title/Abstract]) OR "Central Africa"[Title/Abstract]) OR "Eastern Africa"[Title/Abstract]) OR "Southern Africa"[Title/Abstract]) OR "Western Asia"[Title/Abstract]) OR Asia[Title/Abstract]) OR "Central Asia"[Title/Abstract]) OR "South-eastern Asia"[Title/Abstract]) OR "Caribbean Region"[Title/Abstract]) OR "West Indies"[Title/Abstract]) OR "South America"[Title/Abstract]) OR" Latin America"[Title/Abstract]) OR "Central America"[Title/Abstract]) OR Afghanistan[Title/Abstract]) OR Albania[Title/Abstract]) OR Algeria[Title/Abstract]) OR American Samoa[Title/Abstract]) OR Angola[Title/Abstract]) OR Armenia[Title/Abstract]) OR Azerbaijan[Title/Abstract]) OR Bangladesh [Title/Abstract]) OR Belarus[Title/Abstract]) OR Belize[Title/Abstract]) OR Benin[Title/Abstract]) OR Bhutan[Title/Abstract]) OR Bolivia[Title/Abstract]) OR "Bosnia and Herzegovina"[Title/Abstract]) OR Botswana[Title/Abstract]) OR Brazil[Title/Abstract]) OR Bulgaria[Title/Abstract]) OR "Burkina Faso"[Title/Abstract]) OR Burundi[Title/Abstract]) OR" Cape Verde"[Title/Abstract]) OR Cambodia[Title/Abstract]) OR Cameroon[Title/Abstract]) OR "Central African Republic"[Title/Abstract]) OR Chad[Title/Abstract]) OR China[Title/Abstract]) OR Colombia[Title/Abstract]) OR Comoros[Title/Abstract]) OR" Democratic Republic of Congo"[Title/Abstract]) OR" Congo Republic"[Title/Abstract]) OR" Costa Rica"[Title/Abstract]) OR " Cote d'Ivoire"[Title/Abstract]) OR " Ivory Coast"[Title/Abstract]) OR Cuba[Title/Abstract]) OR "Czech Republic"[Title/Abstract]) OR "Dominican Republic"[Title/Abstract]) OR Croatia[Title/Abstract]) OR Chile[Title/Abstract]) OR Czechoslovakia[Title/Abstract]) OR Cyprus[Title/Abstract]) OR Djibouti[Title/Abstract]) OR Dominica[Title/Abstract]) OR Ecuador[Title/Abstract]) OR Egypt[Title/Abstract]) OR "El Savador"[Title/Abstract]) OR "Equatorial Guinea"[Title/Abstract]) OR Eritrea[Title/Abstract]) OR Ethiopia[Title/Abstract]) OR Fiji[Title/Abstract]) OR Gabon[Title/Abstract]) OR The Gambia[Title/Abstract]) OR Georgia[Title/Abstract]) OR Ghana[Title/Abstract]) OR Grenada[Title/Abstract]) OR Guatamela[Title/Abstract]) OR Guinea[Title/Abstract]) OR " Guinea Bissau"[Title/Abstract]) OR Guyana[Title/Abstract]) OR Haiti[Title/Abstract]) OR Honduras[Title/Abstract]) OR (India [Title/Abstract] OR OR)) OR Indonesia[Title/Abstract]) OR Iran[Title/Abstract]) OR Iraq[Title/Abstract]) OR Jamaica[Title/Abstract]) OR Jordan[Title/Abstract]) OR Kazakhastan[Title/Abstract]) OR Kenya[Title/Abstract]) OR Kiribati[Title/Abstract]) OR "Democratic People Republic of Korea"[Title/Abstract]) OR Kosovo[Title/Abstract]) OR Kyrgyz Republic[Title/Abstract]) OR" Lao DPR"[Title/Abstract]) OR Lebanon[Title/Abstract]) OR Lesotho[Title/Abstract]) OR Liberia[Title/Abstract]) OR Libya[Title/Abstract]) OR Macedonia[Title/Abstract]) OR Madagascar[Title/Abstract]) OR Malawi[Title/Abstract]) OR Malaysia[Title/Abstract]) OR Maldives[Title/Abstract]) OR Mali[Title/Abstract]) OR Marshall Islands[Title/Abstract]) OR Mauritania[Title/Abstract]) OR Mauritius[Title/Abstract]) OR Mexico[Title/Abstract]) OR Micronesia[Title/Abstract]) OR Moldova[Title/Abstract]) OR Mongolia[Title/Abstract]) OR Morocco[Title/Abstract]) OR Mozambique[Title/Abstract]) OR Myanmar[Title/Abstract]) OR Namibia[Title/Abstract]) OR Nepal[Title/Abstract]) OR Nicaragua[Title/Abstract]) OR Niger[Title/Abstract]) OR Nigeria[Title/Abstract]) OR Pakistan[Title/Abstract]) OR Palau[Title/Abstract]) OR Panama[Title/Abstract]) OR" Papua New Guinea"[Title/Abstract]) OR Paraguay[Title/Abstract]) OR Peru[Title/Abstract]) OR Philippines[Title/Abstract]) OR Romania[Title/Abstract]) OR Russian Federation[Title/Abstract]) OR Rwanda[Title/Abstract]) OR Samoa[Title/Abstract]) OR "Sao Tome Principe"[Title/Abstract]) OR Senegal[Title/Abstract]) OR Serbia[Title/Abstract]) OR Sierra Leonne[Title/Abstract]) OR "Solomon Islands"[Title/Abstract]) OR Somalia[Title/Abstract]) OR "South Africa"[Title/Abstract]) OR South Sudan[Title/Abstract]) OR "Sri Lanka"[Title/Abstract]) OR "St Lucia"[Title/Abstract]) OR "St Vincent and the Grenadines"[Title/Abstract]) OR Sudan[Title/Abstract]) OR Suriname[Title/Abstract]) OR Swaziland[Title/Abstract]) OR "Syrian Arab Republic"[Title/Abstract]) OR Tajikistan[Title/Abstract]) OR Tanzania[Title/Abstract]) OR Thailand[Title/Abstract]) OR Timor-Leste[Title/Abstract]) OR Togo[Title/Abstract]) OR Tonga[Title/Abstract]) OR Tunisia[Title/Abstract]) OR Turkey[Title/Abstract]) OR Turkmenistan[Title/Abstract]) OR Tuvalu[Title/Abstract]) OR Uganda[Title/Abstract]) OR Ukraine[Title/Abstract]) OR Uzbekistan[Title/Abstract]) OR Vanuatu[Title/Abstract]) OR Vietnam[Title/Abstract]) OR (West Bank[Title/Abstract] AND Gaza[Title/Abstract])) OR Yemen[Title/Abstract]) OR Zambia[Title/Abstract]) OR Zimbabwe[Title/Abstract]))))  7. ((((((((((((tool*[Title/Abstract]) OR surve*[Title/Abstract]) OR question*[Title/Abstract]) OR measur*[Title/Abstract]) OR instrument*[Title/Abstract]) OR scal*[Title/Abstract]) OR (Surveys and Questionnaires[MeSH Terms])) OR Health Care Surveys[MeSH Terms])) AND (((Patients[MeSH Terms]) OR client*[Title/Abstract]) OR patient*[Title/Abstract])) AND ((((((((((((("Patient Preference"[MeSH Terms]) OR "Patient Outcome Assessment"[MeSH Terms]) OR "Patient-Centered Care"[MeSH Terms]) OR "Patient Satisfaction"[MeSH Terms]) OR patient preference[Title/Abstract]) OR "Patient-Centered Care"[Title/Abstract]) OR "patient experience*"[Title/Abstract]) OR " patient satisfaction"[Title/Abstract]) OR "patient perception*"[Title/Abstract]) OR "patient opinion*"[Title/Abstract]) OR " patient view*"[Title/Abstract]) OR "responsiveness"[Title/Abstract]) OR "service delivery"[Title/Abstract])) AND ((((((("Delivery of Health Care"[Mesh] OR "Quality Indicators, Health Care"[Mesh] OR "Health Care Evaluation Mechanisms"[Mesh] OR "Process Assessment (Health Care)"[Mesh] OR "Outcome Assessment (Health Care)"[Mesh] OR "Quality of Health Care"[Mesh])) OR "health care quality"[Title/Abstract]) OR "Delivery of Health Care"[Title/Abstract]) OR "Outcome Assessment (Health Care)"[Title/Abstract]) OR " Health care evaluation mechanism*"[Title/Abstract]) OR "Quality Indicators,Health Care"[Title/Abstract])) AND (((((((((((((((((((((((((((((((((((((((((((((((((((((((((((((((((((((((((((((((((((((((((((((((((((((((((((((((((((((((((((((((((((((((((((((((((((((((((((((((((("Developing Countries"[Mesh]) OR "low income countr*"[Title/Abstract]) OR "middle income countr*"[Title/Abstract]) OR " low middle income countr*"[Title/Abstract]) OR "Poor countr*"[Title/Abstract]) OR " Northern Africa"[Title/Abstract]) OR "South of the Sahara Africa"[Title/Abstract]) OR "Central Africa"[Title/Abstract]) OR "Eastern Africa"[Title/Abstract]) OR "Southern Africa"[Title/Abstract]) OR "Western Asia"[Title/Abstract]) OR Asia[Title/Abstract]) OR "Central Asia"[Title/Abstract]) OR "South-eastern Asia"[Title/Abstract]) OR "Caribbean Region"[Title/Abstract]) OR "West Indies"[Title/Abstract]) OR "South America"[Title/Abstract]) OR" Latin America"[Title/Abstract]) OR "Central America"[Title/Abstract]) OR Afghanistan[Title/Abstract]) OR Albania[Title/Abstract]) OR Algeria[Title/Abstract]) OR American Samoa[Title/Abstract]) OR Angola[Title/Abstract]) OR Armenia[Title/Abstract]) OR Azerbaijan[Title/Abstract]) OR Bangladesh [Title/Abstract]) OR Belarus[Title/Abstract]) OR Belize[Title/Abstract]) OR Benin[Title/Abstract]) OR Bhutan[Title/Abstract]) OR Bolivia[Title/Abstract]) OR "Bosnia and Herzegovina"[Title/Abstract]) OR Botswana[Title/Abstract]) OR Brazil[Title/Abstract]) OR Bulgaria[Title/Abstract]) OR "Burkina Faso"[Title/Abstract]) OR Burundi[Title/Abstract]) OR" Cape Verde"[Title/Abstract]) OR Cambodia[Title/Abstract]) OR Cameroon[Title/Abstract]) OR "Central African Republic"[Title/Abstract]) OR Chad[Title/Abstract]) OR China[Title/Abstract]) OR Colombia[Title/Abstract]) OR Comoros[Title/Abstract]) OR" Democratic Republic of Congo"[Title/Abstract]) OR" Congo Republic"[Title/Abstract]) OR" Costa Rica"[Title/Abstract]) OR " Cote d'Ivoire"[Title/Abstract]) OR " Ivory Coast"[Title/Abstract]) OR Cuba[Title/Abstract]) OR "Czech Republic"[Title/Abstract]) OR "Dominican Republic"[Title/Abstract]) OR Croatia[Title/Abstract]) OR Chile[Title/Abstract]) OR Czechoslovakia[Title/Abstract]) OR Cyprus[Title/Abstract]) OR Djibouti[Title/Abstract]) OR Dominica[Title/Abstract]) OR Ecuador[Title/Abstract]) OR Egypt[Title/Abstract]) OR "El Savador"[Title/Abstract]) OR "Equatorial Guinea"[Title/Abstract]) OR Eritrea[Title/Abstract]) OR Ethiopia[Title/Abstract]) OR Fiji[Title/Abstract]) OR Gabon[Title/Abstract]) OR The Gambia[Title/Abstract]) OR Georgia[Title/Abstract]) OR Ghana[Title/Abstract]) OR Grenada[Title/Abstract]) OR Guatamela[Title/Abstract]) OR Guinea[Title/Abstract]) OR " Guinea Bissau"[Title/Abstract]) OR Guyana[Title/Abstract]) OR Haiti[Title/Abstract]) OR Honduras[Title/Abstract]) OR (India [Title/Abstract] OR OR)) OR Indonesia[Title/Abstract]) OR Iran[Title/Abstract]) OR Iraq[Title/Abstract]) OR Jamaica[Title/Abstract]) OR Jordan[Title/Abstract]) OR Kazakhastan[Title/Abstract]) OR Kenya[Title/Abstract]) OR Kiribati[Title/Abstract]) OR "Democratic People Republic of Korea"[Title/Abstract]) OR Kosovo[Title/Abstract]) OR Kyrgyz Republic[Title/Abstract]) OR" Lao DPR"[Title/Abstract]) OR Lebanon[Title/Abstract]) OR Lesotho[Title/Abstract]) OR Liberia[Title/Abstract]) OR Libya[Title/Abstract]) OR Macedonia[Title/Abstract]) OR Madagascar[Title/Abstract]) OR Malawi[Title/Abstract]) OR Malaysia[Title/Abstract]) OR Maldives[Title/Abstract]) OR Mali[Title/Abstract]) OR Marshall Islands[Title/Abstract]) OR Mauritania[Title/Abstract]) OR Mauritius[Title/Abstract]) OR Mexico[Title/Abstract]) OR Micronesia[Title/Abstract]) OR Moldova[Title/Abstract]) OR Mongolia[Title/Abstract]) OR Morocco[Title/Abstract]) OR Mozambique[Title/Abstract]) OR Myanmar[Title/Abstract]) OR Namibia[Title/Abstract]) OR Nepal[Title/Abstract]) OR Nicaragua[Title/Abstract]) OR Niger[Title/Abstract]) OR Nigeria[Title/Abstract]) OR Pakistan[Title/Abstract]) OR Palau[Title/Abstract]) OR Panama[Title/Abstract]) OR" Papua New Guinea"[Title/Abstract]) OR Paraguay[Title/Abstract]) OR Peru[Title/Abstract]) OR Philippines[Title/Abstract]) OR Romania[Title/Abstract]) OR Russian Federation[Title/Abstract]) OR Rwanda[Title/Abstract]) OR Samoa[Title/Abstract]) OR "Sao Tome Principe"[Title/Abstract]) OR Senegal[Title/Abstract]) OR Serbia[Title/Abstract]) OR Sierra Leonne[Title/Abstract]) OR "Solomon Islands"[Title/Abstract]) OR Somalia[Title/Abstract]) OR "South Africa"[Title/Abstract]) OR South Sudan[Title/Abstract]) OR "Sri Lanka"[Title/Abstract]) OR "St Lucia"[Title/Abstract]) OR "St Vincent and the Grenadines"[Title/Abstract]) OR Sudan[Title/Abstract]) OR Suriname[Title/Abstract]) OR Swaziland[Title/Abstract]) OR "Syrian Arab Republic"[Title/Abstract]) OR Tajikistan[Title/Abstract]) OR Tanzania[Title/Abstract]) OR Thailand[Title/Abstract]) OR Timor-Leste[Title/Abstract]) OR Togo[Title/Abstract]) OR Tonga[Title/Abstract]) OR Tunisia[Title/Abstract]) OR Turkey[Title/Abstract]) OR Turkmenistan[Title/Abstract]) OR Tuvalu[Title/Abstract]) OR Uganda[Title/Abstract]) OR Ukraine[Title/Abstract]) OR Uzbekistan[Title/Abstract]) OR Vanuatu[Title/Abstract]) OR Vietnam[Title/Abstract]) OR (West Bank[Title/Abstract] AND Gaza[Title/Abstract])) OR Yemen[Title/Abstract]) OR Zambia[Title/Abstract]) OR Zimbabwe[Title/Abstract]))))  Results= 3773 |
| 2. SCOPUS |
| TITLE-ABS-KEY ( "tool*" OR "surve*" OR "questionnaire*" OR "measur*" OR "scal*" OR "instrument*" ) AND TITLE-ABS-KEY ( "patient experience" OR "Client experience" OR "patient opinion*" OR "Client opinion*" OR "patient view*" OR "client view*" OR "Patient Satisfaction" OR "Client Satisfaction" OR "Patient Preference" OR "Client Preference" OR "responsiveness" OR "service delivery" OR "Patient Centered Care" OR "patient centered" OR "client-centered" OR "client Centered Care" OR "patient reported outcome*" ) AND TITLE-ABS-KEY( "Outcome assessment (Health Care)" OR "Process Assessment (Health Care)" OR "Health Care Delivery" OR "health care" OR "health care quality" OR "Health care evaluation mechanism*" ) AND TITLE-ABS-KEY ( "Afghanistan" OR "Albania" OR "Algeria" OR "American Samoa" OR "Angola" OR "Armenia" OR "Azerbaijan" OR "Bangladesh" OR "Belarus" OR "Belize" OR "Benin" OR "Bhutan" OR "Bolivia" OR "Bosnia and Herzegovina" OR "Botswana" OR "Brazil" OR "Bulgaria" OR "Burkina Faso" OR "Burundi" OR "Cabo Verde" OR "Cambodia" OR "Cameroon" OR "Central African Republic" OR "Chad" OR "China" OR "Colombia" OR "Comoros" OR "Democratic Republic of Congo" OR "Congo" OR "Costa Rica" OR "Cote dIvoire" OR "Ivory Coast" OR "Cuba" OR "Djibouti" OR "Dominica" OR "Dominican Republic" OR "Ecuador" OR "Egypt" OR "El Savador" OR "Equatorial Guinea" OR "Eritrea" OR "Ethiopia" OR "Fiji" OR "Gabon" OR "The Gambia" OR "Georgia" OR "Ghana" OR "Grenada" OR "Guatamela" OR "Guinea" OR "Guinea Bissau" OR "Guyana" OR "Haiti" OR "Honduras" OR "India" OR "Indonesia" OR "Iran" OR "Iraq" OR "Jamaica" OR "Jordan" OR "Kazakhastan" OR "Kenya" OR "Kiribati" OR "Democratic Peoples Republic of Korea" OR "Kosovo" OR "Kyrgyz Republic" OR "Lao DPR" OR "Lebanon" OR "Lesotho" OR "Liberia" OR "Libya" OR "Macedonia" OR "Madagascar" OR "Malawi" OR "Malaysia" OR "Maldives" OR "Mali" OR "Marshall Islands" OR "Mauritania" OR "Mauritius" OR "Mexico" OR "Micronesia" OR "Moldova" OR "Mongolia" OR "Morocco" OR "Mozambique" OR "Myanmar" OR "Namibia" OR "Nepal" OR "Nicaragua" OR "Niger" OR "Nigeria" OR "Pakistan" OR "Palau" OR "Panama" OR "Papua New Guinea" OR "Paraguay" OR "Peru" OR "Philippines" OR "Romania" OR "Russian Federation" OR "Rwanda" OR "Samoa" OR "Sao Tome and Principe" OR "Senegal" OR "Serbia" OR "Sierra Leonne" OR "Solomon Islands" OR "Somalia" OR "South Africa" OR "South Sudan" OR "Sri Lanka" OR "St Lucia" OR "St Vincent and the Grenadines" OR "Sudan" OR "Suriname" OR "Swaziland" OR "Syrian Arab Republic" OR "Tajikistan" OR "Tanzania" OR "Thailand" OR "Timor-Leste" OR "Togo" OR "Tonga" OR "Tunisia" OR "Turkey" OR "Turkmenistan" OR "Tuvalu" OR "Uganda" OR "Ukraine" OR "Uzbekistan" OR "Vanuatu" OR "Vietnam" OR "West Bank of Gaza" OR "Yemen" OR "Zambia" OR "Zimbabwe" OR "Developing Countries" OR "Low and Middle Income Countries" OR "Poor countr" OR "Northern Africa" OR "South of the Sahara Africa" OR "Central Africa" OR "Eastern Africa" OR "Southern Africa" OR "Western Asia" OR asia OR "Central Asia" OR "South-eastern Asia" OR "Caribbean Region" OR "West Indies" OR "South America" OR "Latin America" OR "Central America" ) AND ( EXCLUDE ( AFFILCOUNTRY,"United States" ) OR EXCLUDE ( AFFILCOUNTRY,"United Kingdom" ) OR EXCLUDE ( AFFILCOUNTRY,"Australia" ) OR EXCLUDE ( AFFILCOUNTRY,"Canada" ) OR EXCLUDE ( AFFILCOUNTRY,"Italy" ) OR EXCLUDE ( AFFILCOUNTRY,"Germany" ) OR EXCLUDE ( AFFILCOUNTRY,"Netherlands" ) OR EXCLUDE ( AFFILCOUNTRY,"Spain" ) OR EXCLUDE ( AFFILCOUNTRY,"France" ) OR EXCLUDE ( AFFILCOUNTRY,"Switzerland" ) OR EXCLUDE ( AFFILCOUNTRY,"Sweden" ) OR EXCLUDE ( AFFILCOUNTRY,"Belgium" ) OR EXCLUDE ( AFFILCOUNTRY,"Denmark" ) OR EXCLUDE ( AFFILCOUNTRY,"Japan" ) OR EXCLUDE ( AFFILCOUNTRY,"New Zealand" ) OR EXCLUDE ( AFFILCOUNTRY,"Greece" ) OR EXCLUDE ( AFFILCOUNTRY,"Norway" ) OR EXCLUDE ( AFFILCOUNTRY,"Austria" ) OR EXCLUDE ( AFFILCOUNTRY,"Poland" ) OR EXCLUDE ( AFFILCOUNTRY,"Finland" ) OR EXCLUDE ( AFFILCOUNTRY,"Ireland" ) OR EXCLUDE ( AFFILCOUNTRY,"Singapore" ) OR EXCLUDE ( AFFILCOUNTRY,"Portugal" ) OR EXCLUDE ( AFFILCOUNTRY,"Saudi Arabia" ) OR EXCLUDE ( AFFILCOUNTRY,"Czech Republic" ) OR EXCLUDE ( AFFILCOUNTRY,"Chile" ) OR EXCLUDE ( AFFILCOUNTRY,"Slovakia" ) OR EXCLUDE ( AFFILCOUNTRY,"Croatia" ) OR EXCLUDE ( AFFILCOUNTRY,"Hungary" ) OR EXCLUDE ( AFFILCOUNTRY,"Slovenia" ) OR EXCLUDE ( AFFILCOUNTRY,"United Arab Emirates" ) OR EXCLUDE ( AFFILCOUNTRY,"Qatar" ) OR EXCLUDE ( AFFILCOUNTRY,"Uruguay" ) OR EXCLUDE ( AFFILCOUNTRY,"Iceland" ) OR EXCLUDE ( AFFILCOUNTRY,"Estonia" ) OR EXCLUDE ( AFFILCOUNTRY,"Lithuania" ) OR EXCLUDE ( AFFILCOUNTRY,"Palestine" ) OR EXCLUDE ( AFFILCOUNTRY,"Luxembourg" ) OR EXCLUDE ( AFFILCOUNTRY,"Bahrain" ) OR EXCLUDE ( AFFILCOUNTRY,"Cyprus" ) OR EXCLUDE ( AFFILCOUNTRY,"Kuwait" ) OR EXCLUDE ( AFFILCOUNTRY,"Venezuela" ) OR EXCLUDE ( AFFILCOUNTRY,"Barbados" ) OR EXCLUDE ( AFFILCOUNTRY,"Puerto Rico" ) OR EXCLUDE ( AFFILCOUNTRY,"Seychelles" ) OR EXCLUDE ( AFFILCOUNTRY,"Martinique" ) OR EXCLUDE ( AFFILCOUNTRY,"Anguilla" ) OR EXCLUDE ( AFFILCOUNTRY,"Cook Islands" ) OR EXCLUDE ( AFFILCOUNTRY,"Guadeloupe" ) OR EXCLUDE ( AFFILCOUNTRY,"Guam" ) ) AND ( EXCLUDE ( SUBJAREA,"AGRI" ) OR EXCLUDE ( SUBJAREA,"ARTS" ) OR EXCLUDE ( SUBJAREA,"CHEM" ) OR EXCLUDE ( SUBJAREA,"COMP" ) OR EXCLUDE ( SUBJAREA,"EART" ) OR EXCLUDE ( SUBJAREA,"ENGI" ) OR EXCLUDE ( SUBJAREA,"ENVI" ) OR EXCLUDE ( SUBJAREA,"MATH" ) OR EXCLUDE ( SUBJAREA,"PHYS" ) ) AND ( LIMIT-TO ( LANGUAGE,"English" ) )  Results= 2557 |
|  |
| 3. CINAHL |
| S1 (MH "Surveys+") OR (MH "Questionnaires+") OR (MH "Scales")  S2 ( "tool*" OR "surve*" OR " question*" OR "measur*" OR "scal*" OR "instrument*" )  S3 S1 OR S2  S4 ( MH "Patient Centered Care") OR (MH "Patient-Reported Outcomes") OR (MH "Patient Preference") OR (MH "Patient Satisfaction+")  S5 ( "Patient preference" OR "client preference" OR "Patient-Centered Care" OR "Client-Centered Care" OR "patient experience*" OR "client experience*" OR "patient satisfaction" OR "client satisfaction" OR "patient perception*" OR "client perception*" OR "patient opinion*" OR "client opinion*" OR "patient view*" OR "client view*" OR "responsiveness" OR "service delivery" OR "patient reported outcome*" )  S6 S4 OR S5  S7 (MH "Outcomes (Health Care)+") OR (MH "Quality of Health Care+") OR (MH "Health Care Delivery+") OR (MH "Process Assessment (Health Care)+")  S8 ( "health care" OR "health care quality" OR "Health Care Delivery" OR "Process Assessment Health Care" OR "Outcomes (Health Care)  S9 S7 OR S8  S10 (MH "Developing Countries") OR (MH "Low and Middle Income Countries") OR "Poor countr" OR "Northern Africa" OR "South of the Sahara Africa" OR "Central Africa" OR "Eastern Africa" OR "Southern Africa" OR "Western Asia" OR asia OR "Central Asia" OR "South-eastern Asia" OR "Caribbean Region" OR "West Indies" OR "South America" OR "Latin America" OR "Central America" OR "Afghanistan" OR "Albania" OR "Algeria" OR "American Samoa" OR "Angola" OR "Armenia" OR "Azerbaijan" OR "Bangladesh" OR "Belarus" OR "Belize" OR "Benin" OR "Bhutan" OR "Bolivia" OR "Bosnia and Herzegovina" OR "Botswana" OR "Brazil" OR "Bulgaria" OR "Burkina Faso" OR "Burundi" OR "Cabo Verde" OR "Cambodia" OR "Cameroon" OR "Central African Republic" OR "Chad" OR "China" OR "Colombia" OR "Comoros" OR "Democratic Republic of Congo" OR "Congo" OR "Costa Rica" OR "Cote dIvoire" OR "Ivory Coast" OR "Cuba" OR "Djibouti" OR "Dominica" OR "Dominican Republic" OR "Ecuador" OR "Egypt" OR "El Savador" OR "Equatorial Guinea" OR "Eritrea" OR "Ethiopia" OR "Fiji" OR "Gabon" OR "The Gambia" OR "Georgia" OR "Ghana" OR "Grenada" OR "Guatamela" OR "Guinea" OR "Guinea Bissau" OR "Guyana" OR "Haiti" OR "Honduras" OR "India" OR "Indonesia" OR "Iran" OR "Iraq" OR "Jamaica" OR "Jordan" OR "Kazakhastan" OR "Kenya" OR "Kiribati" OR "Democratic Peoples Republic of Korea" OR "Kosovo" OR "Kyrgyz Republic" OR "Lao DPR" OR "Lebanon" OR "Lesotho" OR "Liberia" OR "Libya" OR "Macedonia" OR "Madagascar" OR "Malawi" OR "Malaysia" OR "Maldives" OR "Mali" OR "Marshall Islands" OR "Mauritania" OR "Mauritius" OR "Mexico" OR "Micronesia" OR "Moldova" OR "Mongolia" OR "Morocco" OR "Mozambique" OR "Myanmar" OR "Namibia" OR "Nepal" OR "Nicaragua" OR "Niger" OR "Nigeria" OR "Pakistan" OR "Palau" OR "Panama" OR "Papua New Guinea" OR "Paraguay" OR "Peru" OR "Philippines" OR "Romania" OR "Russian Federation" OR "Rwanda" OR "Samoa" OR "Sao Tome and Principe" OR "Senegal" OR "Serbia" OR "Sierra Leonne" OR "Solomon Islands" OR "Somalia" OR "South Africa" OR "South Sudan" OR "Sri Lanka" OR "St Lucia" OR "St Vincent and the Grenadines" OR "Sudan" OR "Suriname" OR "Swaziland" OR "Syrian Arab Republic" OR "Tajikistan" OR "Tanzania" OR "Thailand" OR "Timor-Leste" OR "Togo" OR "Tonga" OR "Tunisia" OR "Turkey" OR "Turkmenistan" OR "Tuvalu" OR "Uganda" OR "Ukraine" OR "Uzbekistan" OR "Vanuatu" OR "Vietnam" OR "West Bank of Gaza" OR "Yemen" OR "Zambia" OR "Zimbabwe"  S9 S3 AND S6 AND S9 AND S10  Results = 3328 |
