# Supplementary material for: Tools for measuring client experiences and satisfaction with healthcare in low- and middle-income countries: a systematic review of measurement properties
Source: BMC Health Serv Res. 2023 Feb 9;23:133. doi: 10.1186/s12913-023-09129-9 (PMC9909903; doi:10.1186/s12913-023-09129-9)
Supplement: Supplementary file 2 — Additional file 2. Measurement property definitions and appraisal parameters (Terwee et al. 2007). [file 12913_2023_9129_MOESM2_ESM.docx]

**Additional file 2**: **Measurement property definitions and appraisal parameters (Terwee et al. 2007)**

| Domain | Measurement Property | Definition | Criteria for Appraisal of results | |
| --- | --- | --- | --- | --- |
| Reliability | Internal consistency | The degree of the interrelatedness among items | +  ?  **̶** | Cronbach alpha(s) scores ≥0.70 and < 0.95  Unable to score due to missing or unclear information  Criteria for ‘+’ not met |
|  | Test-retest | The ability of the measure to produce similar results in similar conditions | +  ?  **̶** | ICC agreement/weighted Kappa ≥ 0.70 OR ICC consistency/ICC without approach stated/Pearson's r ≥ 0.80 OR unweighted kappa/or kappa without approach stated ≥ 0.80  Not able to score because of unclear or missing information.  Criteria for ‘+’ not met |
|  | Measurement error | The degree to which the scores on repeated measures are close to each other | +  ?  **̶** | MIC ≥ SDC OR MIC outside the LOA OR convincing arguments that agreement  is acceptable  Not able to score because of unclear or missing information  Criteria for ‘+’ not met |
| Validity | Content validity | The degree to which an instrument reflects the theoretical construct to be measured | +  ?  **̶** | Target group and/or experts considered all items to be relevant AND considered the item set to be complete  Not able to score because of unclear or missing information  Criteria for ‘+’ not met |
|  | Construct validity | | | |
|  | Structural validity | The degree to which the scores of the tool adequately reflect the dimensions of the construct being assessed | +  ?  **̶** | Factors should explain at least 50 % of the variance  Explained variance not mentioned  Criteria for ‘+’ not met |
|  | Hypothesis testing | The extent to which the scores of the tool are consistent with pre-formulated hypotheses | +  ?  **̶** | (At least 75% of the results are in accordance with the hypotheses AND, if calculated, the correlation with an instrument measuring the same construct is ≥ 0.50) AND correlations with related constructs are higher than with unrelated constructs if calculated.  Not able to score because of unclear or missing information  Criteria for ‘+’ not met |
|  | Cross-cultural validity | The degree to which the items on a translated version are an adequate reflection of the original version | +  ?  **̶** | The original factor structure is confirmed AND no important DIF found. If only one of these properties are investigated: either the factor structure is confirmed OR no important DIF found.  Not able to score because of unclear or missing information  Criteria for ‘+’ not met |
|  | Criterion validity | The degree to which scores of an instrument reflect the gold standard | +  ?  **̶** | Correlations with chosen gold standard ≥ 0.70, OR AUC ≥ 0.80, OR (specificity AND sensitivity ≥ 80)  Not able to score because of unclear or missing information  Criteria for ‘+’ not met |
| Responsiveness |  | The ability of an instrument to detect changes overtime in construct to be measured | +  ?  **̶** | Correlations of change scores of the target instrument with an instrument measuring the same construct are ≥ 0.40 OR at least 75% of the results are in accordance with the hypotheses OR AUC ≥ 0.70) AND Correlations of change scores of the target instrument with an instrument measuring a related constructs are higher than with unrelated construct if calculated.  Not able to score because of unclear or missing information  Criteria for ‘+’ not met |

Abbreviations: CC: Correlation Coefficient; ICC: Intraclass Correlation Coefficient; MIC: Minimal important change; Pearson’s r: Pearson correlation coefficient; SDC: Smallest Detectable Change.
