# Supplementary material for: Tools for measuring client experiences and satisfaction with healthcare in low- and middle-income countries: a systematic review of measurement properties
Source: BMC Health Serv Res. 2023 Feb 9;23:133. doi: 10.1186/s12913-023-09129-9 (PMC9909903; doi:10.1186/s12913-023-09129-9)
Supplement: Supplementary file 3 — Additional file 3. Axis appraisal results. [file 12913_2023_9129_MOESM3_ESM.docx]

**Additional file 3: Axis appraisal results**

|  | 1. CH-OPSQ | 2. OPEQ | 3. SF-HKEQ | 4. IPSQ | 5. OPREM-CCH | 6. IPREM-CCH | 7. I-PAHC | 8. O-PAHC | 9. PPQ | 10. NIOPDSS | 11. PISQ | 12. PSC | 13. PSS | 14. ERSaPaCE |
| --- | --- | --- | --- | --- | --- | --- | --- | --- | --- | --- | --- | --- | --- | --- |
| Introduction  1. Were the aims/objectives of the study clear? | Y | Y | Y | Y | Y | Y | Y | Y | Y | Y | Y | Y | Y | Y |
| Methods  2. Was the study design appropriate for the stated aim(s)? | Y | Y | Y | Y | Y | Y | Y | Y | Y | Y | Y | Y | Y | Y |
| 3. Was the sample size justified? | N | Y | Y | N | Y | Y | Y | Y | Y | Y | Y | N | Y | N |
| 4. Was the target/reference population clearly defined? (Is it clear who the research was about?) | Y | Y | Y | Y | Y | Y | Y | Y | Y | Y | Y | Y | Y | Y |
| 5. Was the sample frame taken from an appropriate population base so that it closely represented the target/reference population under investigation? | Y | Y | Y | Y | Y | Y | Y | Y | Y | Y | Y | Y | Y | Y |
| 6. Was the selection process likely to select subjects/participants that were representative | Y | Y | Y | Y | Y | Y | Y | Y | Y | DK | Y | Y | Y | N |
| 7. Were measures undertaken to address and categorize non-responders? | N | N | DK | N | N | N | Y | Y | Y | Y | Y | N | N | N |
| 8. Were the risk factor and outcome variables measured appropriate to the aims of the study? | Y | Y | Y | Y | Y | Y | Y | Y | Y | Y | Y | Y | Y | Y |
| 9. Were the risk factor and outcome variables measured correctly using instruments/ measurements that had been trialled, piloted or published previously? | Y | Y | Y | Y | Y | Y | Y | Y | Y | Y | Y | Y | Y | Y |
| 10. Is it clear what was used to determined statistical significance and/or precision estimates? (eg, p values, CIs) | Y | Y | Y | Y | Y | Y | Y | Y | Y | Y | Y | Y | Y | Y |
| 11. Were the methods (including statistical methods) sufficiently described to enable them to be repeated? | Y | Y | Y | Y | Y | Y | Y | Y | Y | Y | Y | Y | Y | Y |
| Results  12. Were the basic data adequately described? | N | Y | Y | Y | Y | Y | Y | Y | Y | Y | Y | Y | Y | Y |
| 13. Does the response rate raise concerns about non-response bias? | DK | N | N | N | N | N | N | N | N | N | N | DK | N | DK |
| 14. If appropriate, was information about non-responders described? | N | N | N | N | N | N | N | N | N | N | N | N | N | N |
| 15. Were the results internally consistent? | Y | Y | Y | Y | Y | Y | N | N | Y | Y | Y | Y | Y | Y |
| 16. Were the results for the analyses described in the methods, presented? | Y | Y | Y | Y | Y | Y | Y | Y | Y | Y | Y | Y | Y | Y |
| Discussion  17. Were the authors’ discussions and conclusions justified by the results? | Y | Y | Y | Y | Y | Y | Y | Y | Y | Y | Y | Y | Y | Y |
| 18. Were the limitations of the study discussed? | Y | Y | Y | Y | Y | Y | Y | Y | N | Y | Y | Y | Y | Y |
| Other  19. Were there any funding sources or conflicts of interest that may affect the authors’ interpretation of the results? | N | N | N | DK | DK | DK | DK | DK | DK | Y | DK | DK | DK | DK |
| 20. Was ethical approval or consent of participants attained? | Y | Y | Y | Y | Y | Y | Y | Y | Y | Y | Y | DK | Y | Y |
| Total score | 15 | 18 | 18 | 16 | 17 | 17 | 17 | 17 | 17 | 17 | 18 | 14 | 17 | 14 |
| KEY: Y; Yes N; No DK; Don’t Know | | | | | | | | | | | | | | |
