# Supplementary material for: Tools for measuring client experiences and satisfaction with healthcare in low- and middle-income countries: a systematic review of measurement properties
Source: BMC Health Serv Res. 2023 Feb 9;23:133. doi: 10.1186/s12913-023-09129-9 (PMC9909903; doi:10.1186/s12913-023-09129-9)
Supplement: Supplementary file 4 — Additional file 4. COSMIN quality assessment results. [file 12913_2023_9129_MOESM4_ESM.docx]

**Additional file 4: COSMIN quality assessment results**

| Instrument | Internal consistency | Measurement Error/ Agreement | Reliability | Content Validity | Structural Validity | Hypotheses  Testing | Cross cultural Validity | Criterion validity | Responsiveness |
| --- | --- | --- | --- | --- | --- | --- | --- | --- | --- |
| 1.CH-OPSQ  CHINA | Fair |  |  | Excellent | Fair |  |  |  |  |
| 2. OPEQ  CHINA | Excellent |  |  | Excellent | Excellent | Fair |  |  |  |
| 3. SF-HKIEQ  CHINA | Poor |  | Fair | Excellent |  |  |  | Fair |  |
| 4.IPSQ CHINA | Fair |  |  | Excellent | Fair | Fair |  |  |  |
| 5. OPREM-CCH CHINA | Excellent |  |  | Excellent | Excellent | Excellent |  | Fair |  |
| 6. IPREM-CCH CHINA | Excellent |  |  | Excellent | Excellent | Excellent |  | Fair |  |
| 7.IPAHC  ETHIOPIA | Excellent |  |  | Excellent | Excellent | Excellent | Fair |  |  |
| 8.OPAHC  ETHIOPIA | Excellent |  |  | Excellent | Excellent | Excellent | Fair |  |  |
| 9.PPQ  INDIA | Excellent |  |  | Excellent | Excellent |  | Poor |  |  |
| 10. NIOPDSS  INDIA | Excellent |  | Poor | Poor | Excellent |  |  |  |  |
| 11. PISQ  PERSIA | Fair |  |  | Excellent | Fair |  |  |  |  |
| 12.PSC EGYPT | Fair |  |  | Poor | Fair |  |  |  |  |
| 13.PSS MIDDLE EAST | Good |  |  | Excellent | Fair |  | Poor |  |  |
| 14. ERSaPaCE  MEXICO | Poor |  | Poor | Poor |  | Poor |  |  |  |
