# Supplementary material for: Tools for measuring client experiences and satisfaction with healthcare in low- and middle-income countries: a systematic review of measurement properties
Source: BMC Health Serv Res. 2023 Feb 9;23:133. doi: 10.1186/s12913-023-09129-9 (PMC9909903; doi:10.1186/s12913-023-09129-9)
Supplement: Supplementary file 5 — Additional file 5. Terwee’s criteria quality assessment results. [file 12913_2023_9129_MOESM5_ESM.docx]

**Additional file 5: Terwee’s criteria quality assessment results**

| Instrument | Internal consistency | Measurement Error/ Agreement | Reliability (Test re test) | Content Validity | Structural Validity | Hypotheses  Testing | Cross cultural Validity | Criterion validity | Responsiveness |
| --- | --- | --- | --- | --- | --- | --- | --- | --- | --- |
| 1.CH-OPSQ  CHINA | + | 0 | 0 | + | + | 0 | 0 | 0 | 0 |
| 2. OPEQ  CHINA | + | 0 | 0 | + | + | ? | 0 | 0 | 0 |
| 3. SF-HKIEQ  CHINA | + | 0 | + | + | 0 | 0 | 0 | + | 0 |
| 4.IPSQ CHINA | + | 0 | 0 | + | + | ? | 0 | 0 | 0 |
| 5.OPREM-CCH CHINA | + | 0 | 0 | + | ? | ? | 0 | - | 0 |
| 6.IPREM-CCH CHINA | + | 0 | 0 | + | ? | ? | 0 | - | 0 |
| 7.IPAHC  ETHIOPIA | - | 0 | 0 | + | ? | + | ? | 0 | 0 |
| 8.OPAHC  ETHIOPIA | - | 0 | 0 | + | ? | + | ? | 0 | 0 |
| 9.PPQ  INDIA | + | 0 | 0 | + | + | 0 | ? | 0 | 0 |
| 10. NIOPDSS  INDIA | + | 0 | - | ? | + | 0 | 0 | 0 | 0 |
| 11. PISQ  PERSIA | - | 0 | 0 | + | ? | 0 | 0 | 0 | 0 |
| 12.PSC EGYPT | + | 0 | 0 | ? | + | 0 | 0 | 0 | 0 |
| 13.PSS MIDDLE EAST | + | 0 | 0 | + | + | 0 | ? | 0 | 0 |
| 14. ERSaPaCE  MEXICO | - | 0 | + | ? | 0 | ? | 0 | 0 | 0 |

+ = Meets test criteria (positive rating); ? = Not able to score due to insufficient information (indeterminate rating) ; - = Does not meet criteria (negative rating) ; 0= No information available
